# End of life care of hospitalized patients with Parkinson disease: a retrospective analysis and brief review

**DOI:** 10.3389/fnagi.2023.1265156

**Published:** 2023-09-06

**Authors:** Sakhi Bhansali, Ekhlas Assaedi, Jeryl Ritzi T. Yu, Nymisha Mandava, Claire Sonneborn, Olivia Hogue, Benjamin L. Walter, Renato V. Samala, Adam Margolius

**Affiliations:** ^1^Neurological Institute, Cleveland Clinic, Cleveland, OH, United States; ^2^Institute for Neurosciences, St. Luke’s Medical Center, Quezon City, Philippines; ^3^University of the East Ramon Magsaysay Memorial Medical Center, Quezon City, Philippines; ^4^Department of Palliative and Supportive Care, Cleveland Clinic, Cleveland, OH, United States

**Keywords:** parkinsonism, Parkinson disease, palliative care, antipsychotic, hospice, DNR

## Abstract

**Background:**

Towards the end of life (EOL), persons with parkinsonism (PwP) have complex needs and can present with unique palliative care (PC) challenges. There are no widely accepted guidelines to aid neurologists, hospitalists, or PC clinicians in managing the symptoms of PwP at EOL. We examined a population of PwP at EOL, aiming to describe trends of in-hospital management and utilization of PC services.

**Methods:**

All PwP admitted to two hospitals during 2018 (*N* = 727) were examined retrospectively, assessing those who died in hospital or were discharged with hospice (EOL group, *N* = 35) and comparing them to the main cohort. Their demographics, clinical data, engagement of multidisciplinary and palliative services, code status changes, invasive care, frequency of admissions, and medication administration were assessed.

**Results:**

Among the EOL group, 8 expired in hospital, and 27 were discharged to hospice. Forty-six percent of EOL patients received a PC consultation during their admission. The median interval from admission to death was 37 days. Seventy-seven percent had a full code status on admission. Compared to hospice patients, those who expired in hospital had higher rates of invasive procedures and intensive care unit transfers (41% vs. 75%, in both variables), and lower rates of PC involvement (52% vs. 25%). The transition of code status change for the EOL group from Full code to Do Not Resuscitate (DNR) occurred at a median 4–5 days from admission. For patients that passed in the hospital, the median days from transition of code status to death was 0(IQR 0–1). Levodopa dose deviations were frequent in both EOL and non-EOL group, but contraindicated medications were infrequently administered (11% in EOL group vs. 9% in non-EOL group).

**Conclusion:**

Our data suggest a low utilization of PC services and delayed discussions of goals of care. More work is needed to raise awareness of inpatient teams managing PwP regarding the unique but common challenges facing PwP with advanced disease. A brief narrative review summarizing the suggested management of symptoms common to hospitalized PwP near EOL is provided.

## 1. Introduction

Parkinson disease (PD) is a slowly progressive neurodegenerative disorder. Following Alzheimer’s dementia, PD is the second leading cause of mortality among the neurodegenerative conditions (Feigin et al., 2019). PD diagnosis is often preceded by years of non-motor symptoms. As the disease progresses, motor symptoms worsen and motor complications, including fluctuations and dyskinesia, may appear. Variable patterns of motor trajectories had been described in PD, but progressive terminal motor decline is common (Poonja et al., 2021). Many motor and non-motor symptoms become treatment-resistant in late-stage PD (Kalia and Lang, 2015). Due to the burden of these symptoms, waning response to dopaminergic therapy and cognitive decline, palliative care (PC) interventions are needed (Richfield et al., 2013; Dawson et al., 2022). A consensus committee perceived PC as an “active holistic care of individuals across all ages with serious health-related suffering due to severe illness and especially of those near the end of life” (Radbruch et al., 2020). There are several suggested models for the delivery of PC to PwP, including primary PC or neuropalliative/specialty PC model (Tarolli and Holloway, 2020; Margolius and Samala, 2022). Primary palliative care should ideally be provided by the patient’s neurologist or family practitioner and should commence at the time of diagnosis. As time progresses, and symptoms become more challenging—such as refractory pain, complex psychiatric symptoms, assistance with conflict resolution in establishing goals of care—a referral to specialty PC or consulting a neuro-palliative physician becomes appropriate. In the inpatient setting, neuro-palliative care can be administered either through inpatient PC services, where patients are admitted for the purpose of managing their symptoms, or through PC consult services that offer guidance to the admitting team on effectively addressing the patients’ symptoms. Hospice is a form of PC, typically delivered by an interdisciplinary inpatient or home-based team, that focuses on care at the end of life (EOL). A prognosis of 6 months or less is commonly required for patients to receive hospice care in the United States (Hui et al., 2013). Accumulating evidence, including from a recent randomized controlled trial, suggests that integrating PC in the care of persons with parkinsonism (PwP) leads to an improved quality of life and reduced symptom burden (Wiblin et al., 2017; Kluger et al., 2019).

Towards the EOL, PwP often have complex needs and can present unique PC challenges (Saleem et al., 2013). Interestingly, quality of life and symptom burden concerns are comparable to end-stage cancer patients (Kluger et al., 2019). However, PC for patients with advanced Parkinson’s disease and related disorders (PDRD) is underutilized and lacks awareness (Safarpour et al., 2015; Akbar et al., 2021). Additionally, identifying patients approaching EOL is often difficult in PwP (Campbell et al., 2010; Hindmarsh et al., 2021). One study estimated that 17.3% of PDRD patients in the United States died in the hospital, as opposed to 4% dying in hospice (Moens et al., 2015). There are no widely accepted guidelines to determine hospice eligibility for this population. Few studies, however, have attempted to identify predictors of mortality and suggested criteria for hospice considerations (Goy et al., 2015; Akbar et al., 2021).

The inpatient care of PwP is complex. Compared to non-PD, hospitalized PwP are more likely to require longer lengths of stay and experience delirium, infections, pressure ulcers, syncope, falls, and adverse drug events (Gerlach et al., 2011). In a 2011 systematic review, poor PD control and complications related to PD treatment were identified as major clinical concerns (Gerlach et al., 2011). Inappropriate administration of dopaminergic medications can cause significant complications (Magdalinou et al., 2007; Campbell et al., 2010).

Two large reviews highlighted the need for guidelines concerning the management of hospitalized PD patients (Aminoff et al., 2011; Gerlach et al., 2011). There are no consensus guidelines to aid neurologists, hospitalists and PC clinicians in managing the symptoms of PwP at EOL. Moreover, few studies have explored the experiences of PwP at EOL. In one study investigating persons with PD who died while in the hospital, only 10% of patients had documented EOL care discussions with their providers and 14% were referred to the palliative care team (Walker et al., 2014). In this study, we examined a population of PwP at EOL who died in a hospital setting or were referred to hospice care prior to discharge. We aimed to describe: (i) their clinical characteristics, (ii) trends of in-hospital medical and surgical management, and (iii) engagement and utilization of specialized PC services and ancillary services. Additionally, we reviewed the literature to summarize strategies for managing hospitalized PwP near the EOL.

## 2. Methods

### 2.1. Study population

Details on the study population and data collection were previously published by Yu et al. (2023). In summary, that study interrogated the inpatient management of PwP. Patients were selected by searching the electronic health records for a past medical history or problem list of PD or parkinsonism. The search included admissions to two Cleveland Clinic sites–Fairview Hospital and Main Campus–for calendar year 2018. Patients diagnosed with primary parkinsonism disorder-IPD and atypical forms of parkinsonism, such as progressive supra nuclear palsy, multiple system atrophy, dementia with Lewy bodies, or cortico-basal syndrome, were included. Patients with drug induced parkinsonism were excluded. The original data set contained 925 hospital admissions from 727 patients over 1 year. For the purpose of the current paper, we queried the dataset for patients who met the following criteria: (i) those who expired during the admission or discharged with hospice care, and (ii) those whose diagnosis of primary parkinsonism was confirmed by a neurologist or a geriatrician. This was done to further confirm the exclusion of secondary parkinsonism, drug-induced parkinsonism, and other neurodegenerative dementias. This group will be referred to as EOL group. Forty-seven charts were reviewed and 35 patients fulfilled the inclusion criteria and were included in the final analysis. All other PwP not meeting the criteria were included in the non-EOL group.

### 2.2. Data collection

For each admission, the following data were collected: demographic data (i.e., age, sex, race, and ethnicity), age-adjusted Charlson Comorbidity Index, nutrition status on admission, and length of stay. These data were extracted from the electronic health record using a custom Structured Query Language script. The following additional data were collected whenever available: PD duration, days from discharge to death, number of admissions and emergency room (ER) visits in the year preceding their last hospital admission, code status upon last admission, changes to code status during last admission, caregivers’/patients’ discharge goals, rehabilitation team’s impression of disposition upon admission, final discharge dispositions, utilization of allied health services, such as physical therapy (PT), occupation therapy (OT) and speech therapy (ST), nutrition status, PC team involvement, intensive care unit (ICU) admission, and invasive procedures. The latter was defined as any procedure requiring general anesthesia, including bronchoscopy, biopsies, intubations, hernia repair, palliative ERCP, nephrostomy tube placement, percutaneous endoscopic gastrostomy (PEG) tube insertion, and advanced PD therapy management (DBS, levodopa intestinal gel). The severity of malnutrition was graded based on nutritionists’ evaluation, assessing a composite of subcutaneous fat loss, muscle loss, and functional capacity (White et al., 2012; Phillips, 2014). With regards to code status, “Do Not Resuscitate Comfort Care” (DNR CC) is defined here as no cardiopulmonary resuscitation effort or intubation and the provision of comfort care. This data was extracted via manual chart review by two of the authors (SB, EA).

**Figure 1:**
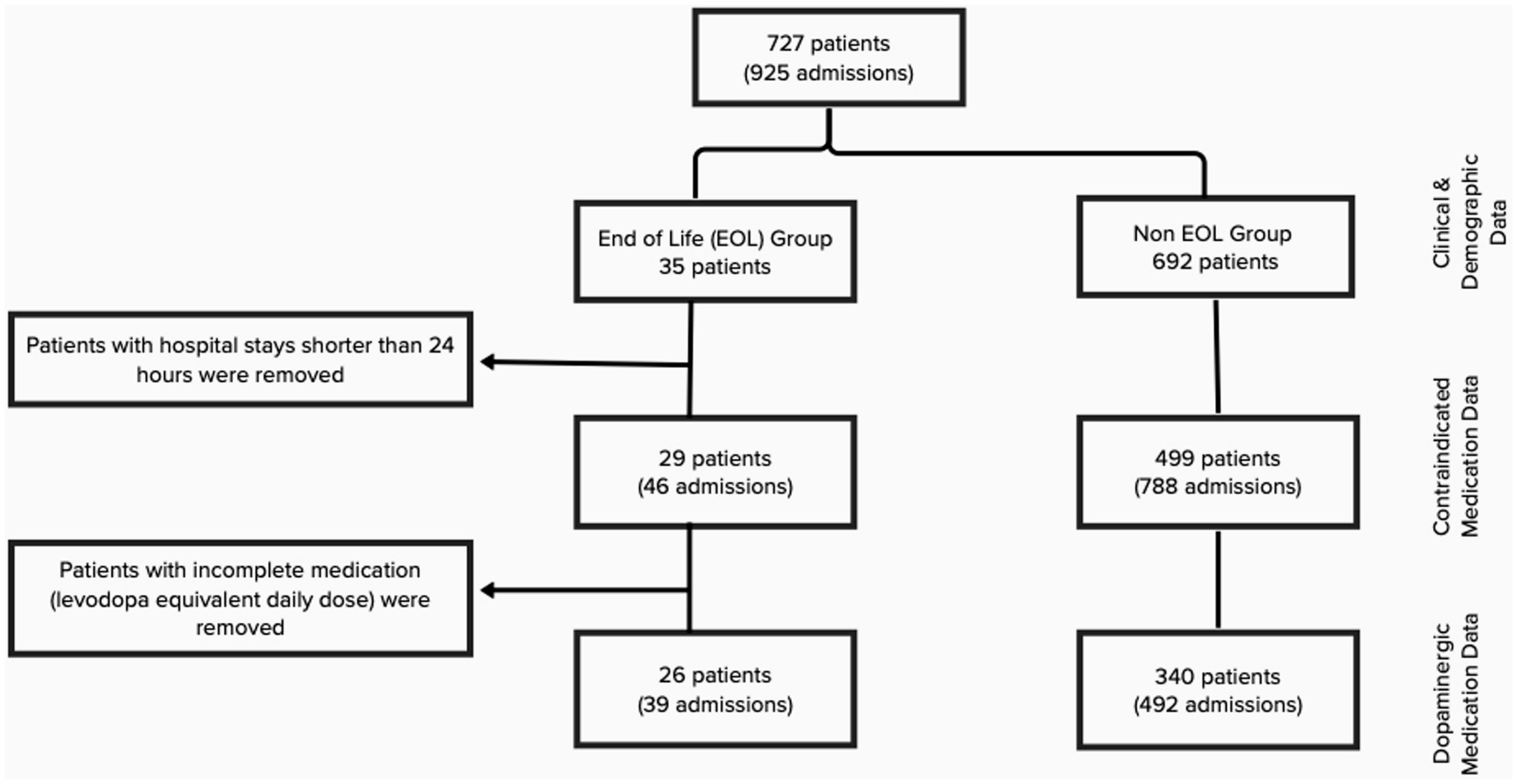
Flowchart of patients and hospital visits included in different stages of the study.

Medication information was reviewed, including levodopa equivalent daily dose (LEDD), deviation from LEDD during admission, and administration of contraindicated medications. Search for contraindicated medications included the following: (1) typical antipsychotics: chlorpromazine, fluphenazine, haloperidol; (2) atypical antipsychotics: risperidone, olanzapine, ziprasidone, aripiprazole, lurasidone, paliperidone, brexpiprazole, asenapine, and (3) antiemetics: metoclopramide, prochlorperazine, promethazine. A manual chart review was conducted by a study team member (JY) to record patients’ time-critical antiparkinsonian outpatient medication regimen. These were defined as products containing levodopa. Patients with available medication data and hospital stays longer than 24 h were reviewed for administration of contraindicated medications (835 hospitalizations, 528 patients). Of the dataset of hospital stays greater than 24 h, 366 patients (531 hospitalizations) had complete medication data allowing for LEDD calculation and analysis of deviations from home regimen (Figure 1).

### 2.3. Statistical analysis

Categorical variables were described in frequencies and percentages. Continuous variables were reported in mean, and standard deviation if normally distributed and in median and interquartile range if skewed. Descriptive statistics were calculated separately for EOL and non-EOL group. Data analysis was carried out using SAS Studio version 3.7.

## 3. Results

### 3.1. Demographics of the general inpatient cohort and EOL cohort

Thirty-five PwP at EOL were reviewed. Baseline demographic and some clinical characteristics were compared to 692 patients with parkinsonism who did not meet inclusion criteria (non-EOL group; Table 1). Compared to non-EOL group, patients in EOL group were older (mean of 80 years vs. 76 years) and had a higher age-adjusted Charlson index score (mean of 9.1 ± 2.8 vs. 7.1 ± 3.3). Both groups had a higher proportion of males compared to females. Date of death was known for 34 patients in EOL group (97%) and 218 (32%) patients in non-EOL group. Among deceased patients, the median interval from hospital admission to death was 37 days for patients in EOL group and 425 days in non-EOL.

**Table 1:** Patients’ demographic and basic clinical characteristics.

Variable	EOL group (*n*=35)	Non-EOL group (*n*=692)
**Gender**		
Female	10 (28.6)	294 (42.5)
Male	25 (71.4)	398 (57.5)
**Race**		
White	25 (75.8)	576 (83.2)
Black	7 (17.2)	69 (10.0)
Multiracial/multicultural	3 (6.9)	15 (2.2)
Asian	0 (0)	8 (1.2)
Other	0 (0)	7 (1.0)
Declined, unavailable or unknown	0 (0)	17 (2.5)
Age, mean (SD)	80.2 (9.9)	75.5 (12.1)
**Known death after discharge**		
Yes	34 (97.1)	218 (31.5)
No	1 (2.9)	474 (68.5)
Among deceased: the number of days from discharge to death, median (IQR)	37 (10–136.5)	425 (128–865)
**Have deep brain stimulation**		
Yes	2 (5.7)	79 (11.4)
No	33 (94.3)	613 (88.6)
Charlson index score, median (IQR)	4 (2–7)	2 (1–5)
Age-adjusted Charlson index score, mean (SD)	9.1 (2.8)	7.1 (3.3)

Values presented represent frequency (%), except where otherwise noted. *N* refers to the number of patients. EOL group includes patients with parkinsonism at the end of life. Non-EOL group includes the remainder of patients with the parkinsonism cohort.

### 3.2. Experience of inpatient PwP at EOL

Only 46% received PC consultation (Table 2). Speech therapists, nutritionists, and physical or occupational therapists were involved in the care of 31, 57, and 51% of patients, respectively. Hospitalizations in the year preceding death were frequent with a median of four admissions. Nearly half of the patients (49%) in the EOL cohort underwent invasive procedures, including intubation, nephrostomy tube placements, bowel surgery, biopsies, and bronchoscopies. Forty nine percent of patients received ICU-level care during their admission. Seventy seven percent of patients had a full code or presumed full code status on admission and 23% had a DNR CC code status. During their hospital stay, code status was changed to DNR CC for all the full code/presumed full code patients, except one patient for whom code status was not clearly documented at the time of discharge. At the time of admission, disposition goals as determined by family or patient preferences and by admitting providers were explicitly stated for 22 and 23 patients, respectively. Twenty percent of patients/families expected a discharge to a rehabilitation or skilled nursing facility, while 31% expected a discharge to home. Eventually, the largest number of patients were discharged to hospice facilities or inpatient hospice (40%), followed by home with hospice care (37%). Eight patients (23%) died in the hospital.

**Table 2:** Clinical characteristics of PwP at EOL.

Variable	EOL group (*N* = 35)
Parkinsonism duration in years, median (IQR)*	7 (4–9)
Number of hospitalizations in the last year, median (IQR)	4 (2–6)
Number of ED visits in the last year, median (IQR)	1 (0–2)
**Code status on admission**	
Full code/presumed full code	27 (77.1)
DNR CC	8 (22.9)
**Had consult with diet/nutrition team**	
Yes	20 (57.1)
No	15 (42.9)
**Had consult with physical/occupational therapy team**	
Yes	18 (51.4)
No	17 (48.6)
**Had consult with speech therapy team**	
Yes	11 (31.4)
No	24 (68.6)
**Had consult with palliative care team**	
Yes	16 (45.7)
No	19 (54.3)
**Had invasive procedure performed**	
Yes	17 (48.6)
No	18 (51.4)
Malnutrition	5 (14.3)
Mild	9 (25.7)
Moderate	9 (25.7)
Severe	12 (34.3)
NA	–
**ICU admission/transfer**	
Yes	17 (48.6)
No	18 (51.4)
**Initial disposition goal by caregiver/patient**	
Acute rehab or skilled nursing facility or extended care facility	7 (20)
Hospice	4 (11.43)
Home	11 (31.43)
NA	13 (37.14)
Initial disposition goal as judged by admitting providers**	
Acute rehab or skilled nursing facility or extended care facility	16 (45.7)
Hospice	4 (11.4)
Home	3 (8.6)
NA	12 (34.3)
**Patient’s final disposition**	
Home with hospice	13 (37.1)
Hospice facility or inpatient hospice	14 (40.0)
Expired	8 (22.9)

Values presented represent frequency (%), except where otherwise noted. *N*=35. Not applicable refer to hospital visits in which patients with missing values; EOL, end of life; ED, emergency department, DNR-CC, Do Not Resuscitate Comfort-Care order; DNI, Do Not Intubate; ICU, intensive care unit. *Disease duration data was available for 28 out of 35 patients. **Patients’/caregiver’s initial disposition goals and providers estimated disposition were available for 23 patients and 22 patients, respectively.

Among PwP at EOL, we compared those who expired in hospital vs. those discharged with hospice care. Average age (82 vs. 81), median PD duration (7 vs. 6 years), and median Charlson Comorbidity Score (5 vs. 4) were relatively similar between the two groups. The group of patients that expired in the hospital had a higher percentage of invasive procedures and ICU admissions compared to the hospice group (75% vs. 41%, respectively for both variables). The hospice group was more likely to receive a palliative care consult (52% vs. 25%). The transition from Full Code to DNR CC for both groups occurred at a median of 4.5(IQR 1.5–9) days and 5(IQR 2–10) days, respectively. Unfortunately, death occurred at a median of 0 days from transition to comfort care for patients that expired in the hospital. Out of 27 patients that were initially full code, 6 passed on the same day of the code status change (Table 3).

**Table 3:** Clinical characteristics of patients who died in hospital versus discharged to hospice care.

Variable	Hospice care (*N*=27)	Expired (*N*=8)
Age, mean (SD)	80 (10.5)	80.6 (8.2)
Hospital stay duration, median (IQR)	6 (3–11)	2 (1–9.5)
Parkinson’s disease duration, median (IQR)	6 (4–9)	7 (6–12)
**Whether the patient had an invasive procedure or not**		
Yes	11 (40.7)	6 (75)
**Whether the patient had a palliative care consult or not**		
Yes	14 (51.9)	2 (25)
**Whether the patient had a speech consult or not**		
Yes	9 (33.3)	2 (25)
**Whether the patient had an ICU admission or not**		
Yes	11 (40.7)	6 (75)
The number of hospital admissions, median (IQR)	4 (2–7)	3 (2.5–4.5)
The number of emergency department visits, median (IQR)	1 (0–2)	0 (0–0.5)
**Code status upon admission**		
Full code or presumed full code	21 (77.8)	6 (75)
DNR CC	6 (22.2)	2 (25)
Duration between DOA to DNR CC in days, median (IQR)	2.5 (0–5)	2 (0–9)
Duration between DNR CC to date of death, median (IQR)	16 (3–35)	0 (0–1)
Charlson Comorbidity index, median (IQR)	4 (2–6)	4.5 (2.5–8)

Values presented represent frequency (%), except where otherwise noted. DNR-CC, Do Not Resuscitate Comfort-Care order; DNI, Do Not Intubate; ICU, intensive care unit, DOA, date of admission.

### 3.3. Medication administration trends in inpatient PwP cohort

With regards to medication administration trends, both groups were subject to frequent LEDD deviations and underdosing from their home regimen while being inpatient (Table 4). Eighty-five percent of hospitalizations in EOL group involved at least one day of LEDD deviation. The largest LEDD deviation was higher for the EOL group (300 mg vs. 147 mg), but missed doses were infrequent in both groups. Eighty five percent of admissions of patients in the EOL group had LEDD. In the EOL group, 80% of admissions had atleast 1 day of levodopa underdosing, 10% had atleast 1 day of levodopa overdosing. The median number of missed doses of levodopa was 1 in both the EOL and non-EOL group. The frequency of contraindicated medications administration was 11% in EOL group and 9% in non-EOL group (Table 5). Both groups received contraindicated medications for a median of 2 days during their hospitalizations. Haloperidol and olanzapine were the most frequently administered medications in both cohorts. While metoclopramide was never given in the EOL group, patients in 21% of hospitalizations in non-EOL group received it during their stay. Among patients in EOL group, three had pre-existing advanced therapies. One patient had a levodopa intestinal gel pump placed *in-situ*, which was not actively used due to malfunction, and two had an active Deep Brain Stimulation (DBS) device placed. One of them died acutely due to intracranial hemorrhage, presumed unrelated to the device. The other patient had an active stimulation upon transfer to hospice. The admitting hospitalist team followed the hospice team’s advice to maintain the device on.

**Table 4:** Patterns of parkinsonism medication administration during hospitalizations.

Variable	EOL group (*n* = 39)	Non-EOL group (*n* = 492)
Length of hospital stay in days, median (IQR)	6.3 (3.3–10.9)	3.7 (1.8–7.4)
Patient's LEDD from their home regimen, median (IQR)	600 (300–800)	600 (300–950)
Full hospital days (not including admission and discharge days), median (IQR)	5 (2–8.5)	2 (1–6)
Number of levodopa doses per day, median (IQR)	4 (3–5)	4 (3–4)
**Whether the patient had at least one day with an LEDD overdose during stay**		
Yes	4 (10.3)	94 (19.1)
No	35 (89.7)	398 (80.9)
**Whether the patient had at least one day with an LEDD underdose during the stay**		
Yes	31 (79.5)	275 (55.9)
No	8 (20.5)	217 (44.1)
**Whether the patient had at least one day with an LEDD deviation during the stay**		
Yes	33 (84.6)	330 (67.1)
No	6 (15.4)	162 (32.9)
Largest daily LEDD deviation, median (IQR)	300 (100–560)	146.5 (0–394.5)
Number of levodopa doses missed in the hospital, median (IQR)	1 (1–2)	1 (0–1)
**Whether the patient had any days with a missing levodopa dose**		
Yes	16 (41.0)	127 (25.8)
No	23 (59.0)	365 (74.2)

For EOL group, *N* = 39 hospitalizations pertaining to 26 unique patients. For non-EOL group, *N* = 492 hospitalizations pertaining to 340 unique patients. Values presented represent frequency (%), except where otherwise noted. LEDD, levodopa equivalent daily dose.

**Table 5:** Frequency of contraindicated medications administration across hospital visits.

Variables	EOL group (*n* = 46)	Non EOL group (*n* = 788)
Contraindicated medications were given*	5 (10.9)	67 (8.5)
Number of days during the hospital visit when at least one contraindicated medication was administered*	2 (1–4)	2 (1–5)
Among hospital visits where contraindicated medications were given**		
Metoclopramide	0 (0)	14 (20.9)
Aripiprazole	1 (20)	9 (13.4)
Haloperidol	4 (80)	35 (52.2)
Risperidone	1 (20)	5 (7.5)
Olanzapine	2 (40)	14 (20.9)
Promethazine	0	4 (6.0)
Paliperidone	0	1 (1.5)

Values presented represent frequency (%). *Values pertain to 46 visits (29 unique patients) involving EOL group and 788 visits (499 unique patients) involving non-EOL group. **Values pertain to 5 visits (5 unique patients) in EOL group and 67 visits (58 unique patients) in non-EOL group; values represent ‘yes’ responses, within each cohort, for each medication out. This table includes hospital visits of patients who have medication data and with hospital stays greater than 24 h.

## 4. Discussion

In this retrospective study, we assessed the experience of PwP at EOL who died while in the hospital or were discharged with hospice, evaluating their clinical characteristics, trends of dopaminergic medication use, and interaction with palliative and medical services. Our population of PwP at EOL had a median interval of 37 days separating admission to death. Despite being older, having multiple comorbidities, and having frequent admissions in the last year of life, the majority of patients did not receive a PC consultation. During their admission, invasive procedures were frequent and admission to ICU were not uncommon. At the time of admission, patient/family and providers’ expectations on disposition goals were not aligned and the majority had a full code/presumed full code status. Most patients were eventually discharged to home or inpatient hospice. When code status was changed, it occurred at a similar median number of days in the hospice vs. inpatient expired population group (4.5 and 5 days, respectively). The median number of days from this transition to death was 0 in the patient group that expired in the hospital.

Our findings suggest a low utilization rate of PC resources. Discussions of goals of care and involvement of PC services were delayed. This was observed more often in the group that expired in the hospital, which also received more invasive care. Our findings are in line with a prior study which emphasized the underutilization of PC resources for PwP at EOL (Nimmons et al., 2020). Lower awareness from the primary inpatient team of the available palliative resources and the appropriateness of PC referral might be one possible explanation. It is possible that patients could not have had palliative care/hospice discussions due to more critical and urgent medical concerns. In such scenarios, patients and family members may benefit from early recognition of declining course and need for goals discussion. We hope that the narrative review that accompanies this article gives readers an overview of providing primary palliative care and knowing when to seek specialist help.

Moreover, prognostication is often difficult in PD especially with the lack of uniformly accepted criteria for PC or hospice referral. In a study investigating a cohort of patients who died of cancer or non-cancer illness and had received PC, more patients with chronic organ failure and dementia had received PC 30 days or less before death relative to cancer patients (Quinn et al., 2021). Earlier involvement of palliative-oriented care may have facilitated an earlier discussion of advance care planning. A Parkinson Disease Quality Measurement Set, published by the American Academy of Neurology in 2016, recommended an annual review of advance directives, but it is unclear how widely this is implemented. Estimated rates of advance directives completion among PwP were reported to vary from 68 to 95% (Kwak et al., 2014; Tuck et al., 2015; Kluger et al., 2018; Lum and Kluger, 2020). The present study did not assess this directly though the rate is postulated to be lower given that the majority of patients at EOL had a full/presumed full code status upon last admission. Similar findings were seen in another study which described that advanced care planning for many patients with PD started as a response to a crisis event like a hospitalization (Nimmons et al., 2020). Our patients who were discharged to hospice had received a PC consultation more frequently. On the other hand, the patients who expired in the hospital, with higher rates of ICU transfers and invasive procedures, were observed to have less frequent interactions with PC services. While the sample was not felt to be powered enough to assess for correlations, previous research had shown that in-hospital PC involvement influenced discharge disposition and improved quality of life (Brody et al., 2010).

In addition to frequent admissions over the last year of life, majority of patients in the EOL group were malnourished and had several comorbid conditions. This is in agreement with other studies showing that recurrent hospitalizations and ED visits increase with longer disease duration (Factor and Molho, 2000; Klein et al., 2009). Some of these factors are among the suggested criteria for triggering hospice referral (Akbar et al., 2021; Centers for Medicare and Medicaid Services, 2023). These trends should be noticed and could be considered as “red flags,” triggering the initiation of advance care planning conversations and/or referral to PC programs/resources.

Lastly, with data from over 500 admissions, LED deviations were frequent among admitted PwP, whether or not at EOL. Contraindicated medications were infrequently administered (11 and 9% in EOL and non EOL group; respectively) in both groups. Patients at EOL were more prone to LEDD compared to the Non EOL group (85% vs. 67%). While differences in the group size did not permit comparison studies, underdosing was more common than overdosing in both groups. While previous studies have reported missed and delayed doses as common errors in administering levodopa (Martinez-Ramirez et al., 2015), these errors were uncommon in this cohort. There could be several reasons why admitting teams were underdosing levodopa, such as the unavailability of a specific strength of levodopa, lack of awareness about the need for strict adherence to the home levodopa regimen, and misconceptions about levodopa being a common cause of neuropsychiatric manifestations. However, administering anti-dopaminergic agents to a particularly vulnerable group suggests more work is needed to enhance the awareness of inpatient teams caring for PwP of potential harm. Other reports of medication errors during admissions ranged between 20 and 50% (Lertxundi et al., 2017; Lance et al., 2021). Such errors had been linked to prolonged hospital stays and increased risk of readmissions (Martinez-Ramirez et al., 2015; Shahgholi et al., 2017).

Several limitations are noted here. As a retrospective study, which partly relied on automatic data retrieval from electronic medical records, the design had its inherent biases. A large number of patients had to be excluded from the levodopa deviations analysis for being admitted for less than 24 h or missing an outpatient regimen. Thus, the cohort may or may not be a representative of the inpatient PwP population. Previous discussions about advanced care planning and medical directives have been shown to increase the utilization of hospice services and reduce hospitalizations (Lum et al., 2019). This may have influenced medical decisions for some patients in the end-of-life (EOL) cohort. This study focused solely on the inpatient course of these patients, and this data wasn’t consistently available for all patients and was not collected. Additionally, the EOL group was small in size, limiting the ability to conduct meaningful comparative analyses to the main cohort.

In summary, this inpatient retrospective study of PwP highlights areas of concern which may affect the quality of life of PwP in their last days. There is an unmet need to expand advanced care planning discussions, particularly in the outpatient setting. More work is needed to raise awareness of inpatient teams managing PwP regarding the unique, but common, challenges facing PwP with advanced disease, their vulnerability to certain medication omissions/administrations, and the value of involving specialty PC and/or movement disorders services. Future prospective studies are needed to assess PwP, in the outpatient and inpatient settings, aiming to assess the integration of movement disorders and PC services, and enhancing the recognition of those who might benefit from earlier facilitation of such resources.

## 5. Review of management recommendations for inpatient PwP at EOL

The following sections will review available literature pertaining to the care of PwP closer to EOL. We highlight pertinent aspects of the inpatient management of common symptoms of advanced PD at EOL. It is meant to aid trainees, hospitalists, and providers in hospitals, nursing homes, and hospice agencies that frequently take care of PwP, and emphasizes areas in which managing PwP might differ from usual PC symptom management. For simplicity, we will review suggested management which may be applicable to the majority of PwP at EOL (Group A). When necessary, specific management points applicable to patients with shorter life expectancy (days to weeks) is provided as Group B.

### 5.1. Palliative care or hospice referral

**Figure 2:**
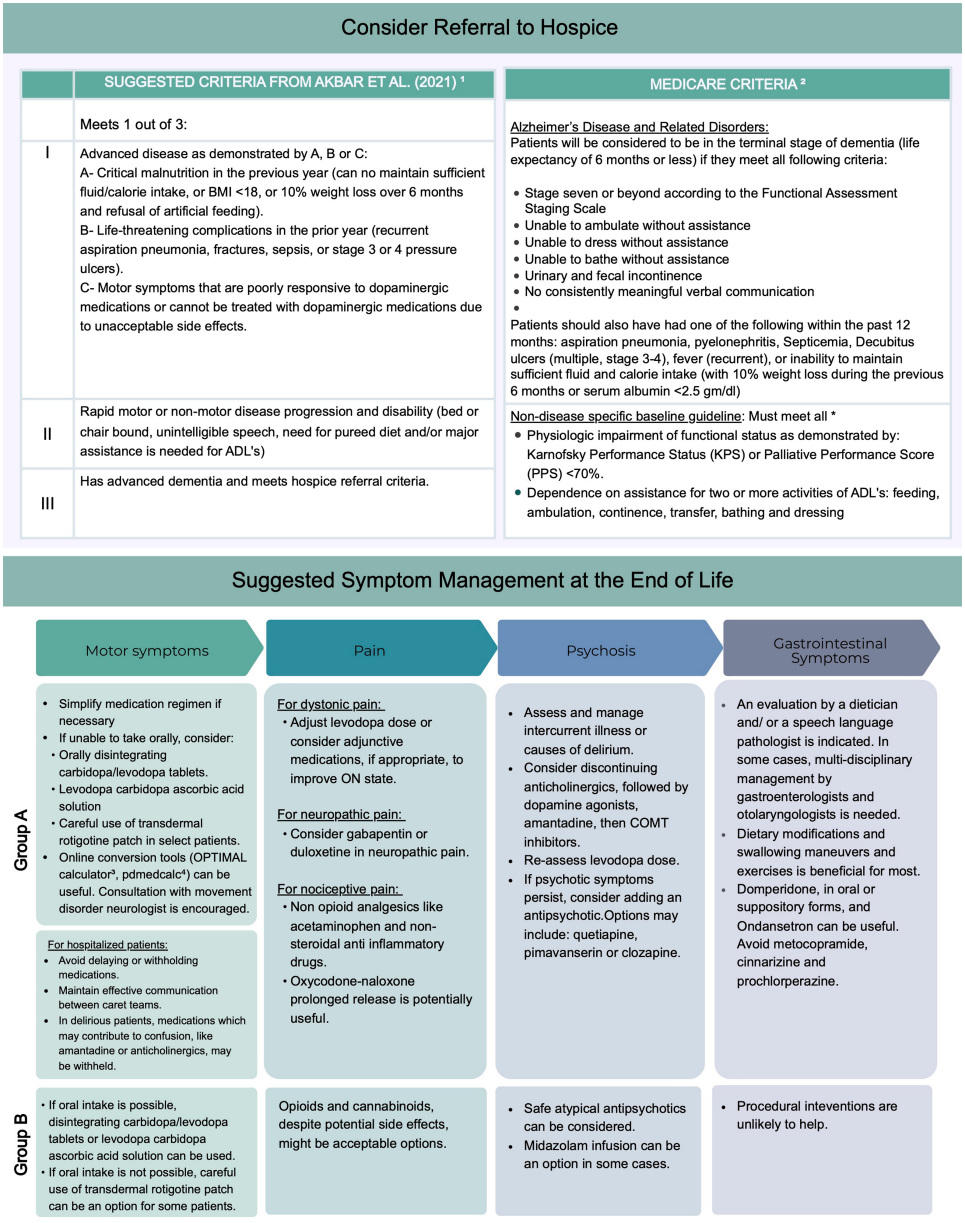
Suggested criteria for hospice referral and suggested outline for management of common symptoms at end of life. Group 1 includes recommendations that apply to the majority of patients with parkinsonism receiving palliative care at end of life. Group 2 includes suggestions that apply to more frail patient anticipated to have shorter life expectancy (Akbar et al., 2021)^1^; (Centers for Medicare and Medicaid Services, 2023)^2^, (Fisher et al., 2017)^3^. ADLs, activities of daily living.

There are no guidelines to indicate when specialty palliative care may be needed for people with Parkinson’s (PwP). Complex symptom management and challenging discussions regarding end-of-life care and advanced care planning may be common reasons for referral or consultation. Recent studies have highlighted specific symptoms or time points in the disease course that may be used as triggers for palliative care referral. These triggers include a significant decrease in functional capacity or caregiver strain, discussions about feeding tubes, distressing psychiatric symptoms, and communication issues with families (Boersma et al., 2014; Creutzfeldt et al., 2016). That being said, there are several barriers that contribute to the underutilization of palliative care for people with Parkinson’s. Underestimating the emotional impact of being diagnosed with PD, insufficient time allocated for advanced care planning, lack of clear responsibilities and roles in introducing palliative care, limited resources, high workloads, and limited communication between healthcare services are some of the common barriers identified that contribute to the underutilization of palliative care in this population (Vaughan and Kluger, 2018; Lennaerts et al., 2019). Neurologists often inform PwP that they will die with PD, not from it. While it is a chronic condition, the age-adjusted mortality ratio is higher in this population, and the leading causes of death are related to complications of PD. Like PC, there are no consensus guidelines to help identify PwP who would benefit from hospice care (Chen et al., 2023). However, a number of experts have suggested recommendations to guide the transition to PC (Goy et al., 2015; Akbar et al., 2021; Margolius and Samala, 2022). Goy et al. suggest that weight loss and shifts in dopaminergic medication prescription trends–reflecting that medication benefits no longer outweigh side effects risk–might be important factors signifying the need to consider hospice referral (Goy et al., 2015). Akbar and colleagues provided a comprehensive list of criteria to determine hospice eligibility (Figure 2). In addition to identifying potential candidates for hospice, it is important to explore patient’s and family’s goals and wishes and ascertain if hospice is in line with these goals. Familiarity with local eligibility criteria (Vaughan and Kluger, 2018; Centers for Medicare and Medicaid Services, 2023) is necessary, though strict adherence to such criteria might hinder the delivery of care.

### 5.2. Managing common symptoms in advanced PD

#### 5.2.1. Motor symptoms

##### 5.2.1.1. Group A

It is advisable to consider simplifying medication regimens to reduce medication pill burden, risk of interactions, and side effects (Aminoff et al., 2011). With disease progression, tolerance to trihexyphenidyl, amantadine, and dopamine agonists is reduced and discontinuing these medications is recommended (Friedman and Factor, 2000). Risk of daytime somnolence, neuropsychiatric, and autonomic side effects become more prominent. Moreover, the dose of dopaminergic medications may need to be revised as non-motor symptoms predominate with disease progression.

When PwP are hospitalized, admitting teams are advised to continue their dopaminergic medications following their home regimen. Following the precise timing of medication administration is necessary since advanced motor fluctuations are common. Care should be taken to avoid delays in medication administration. Effective communication is vital between involved medical teams including emergency physicians, consulting teams and nurses. Parkinsonism-hyperpyrexia syndrome is a rare but life-threatening condition which can occur when dopaminergic medications are rapidly reduced or stopped (Apetauerova et al., 2021). When admitted patients are delirious and workup is underway to explore delirium causes, medications which may contribute to confusion, like amantadine, may be withheld. When a medication is non-formulary for an inpatient pharmacy, patient’s own supply can be used. Alternatively, consultation with a movement disorders neurologist is encouraged. If the need arises to switch from one agent to another, a levodopa equivalent dose (LED) calculator can be a helpful tool. A recent consensus paper from the International Parkinson and Movement Disorders Society had provided updated recommendations on LED calculation (Jost et al., 2023). LED conversion formulae are, however, primarily designed to inform research and their development is limited by the lack of sufficient trials informing dose equivalency.

A frequently encountered scenario is medication administration interruptions due to concerns about swallowing safety or decreased level of consciousness. When PwP lack a safe oral route temporarily, the following options can be considered.

Orally disintegrating carbidopa/levodopa tablet (ODT): the tablets quickly dissolve and can be swallowed with saliva. Unlike sublingually absorbing tablets, these tablets are absorbed in the lower GI tract similar to normal tablets, and are better tolerated in patients with dysphagia especially because it does not require water to swallow it (Morgan and Sethi, 2005; Nausieda et al., 2005). ODT and immediate release carbidopa-levodopa (IR CL) tablets have a similar time to action and duration of action. However, Tmax is achieved faster in ODT compared to IR CL tablets (Ondo et al., 2010). ODT may not be consistently absorbed in patients with ileus. In patients with poor gut motility and severe dysphagia, subcutaneous (SC), sublingual, or transdermal options offer a better drug bioavailability.

Levodopa carbidopa ascorbic acid solution (LCAS): LCAS can be an option for patients with advanced motor fluctuations and limited tolerance to adjunctive medications, patients with dysphagia to pills, or patients requiring nasogastric (NG) feeding (Yang et al., 2017; Sung et al., 2023). One Suggested LCAS recipe is dissolving ten IR CL tablets (100/25 mg) in 1 L of water with ascorbate (2000 mg/L). One ml of LCAS was equivalent to 1 mg of levodopa carbidopa (Sung et al., 2023). The solution can be administered at 1–2-h intervals based on their home LED.

Transdermal rotigotine patch: In cases of poor gastric motility in which patients are not amenable to NG tube placement, transdermal access is the next best strategy. Avoiding inappropriate dosing is necessary especially in patients with dementia. One retrospective study evaluated inpatient use of the rotigotine patch and found that 10% patients had new or worsening hallucinations, and 24% had new or worsening delirium (Ibrahim et al., 2021). The OPTIMAL calculator 2 is publicly accessible and helps calculate rotigotine patch doses based on their prior PD medications (Ibrahim et al., 2021). As part of an initiative to improve hospital safety, the Parkinson Foundation had published a report highlighting the inherent risks of inpatient care(Amodeo et al., 2023). The report includes an NPO protocol summarizing temporary dopaminergic substitutions options.

Some advanced therapies for motor complications can be considered for selected PwP. These include levodopa carbidopa intestinal gel (LCIG) and apomorphine. While they can be utilized for those who had been initiated on these agents previously and temporarily lack safe oral access, initiating such therapies is often not feasible for newly admitted patients for other reasons. Additionally, LCIG initiation is unlikely beneficial in hospice-eligible patients. The guidelines of the European Academy of Neurology and the European section of Movement Disorders Society (EAN-ES MDS) for invasive therapies suggest that clinicians consider offering LCIG to eligible patients with motor fluctuations that are not satisfactorily controlled with oral medications. Based on the results of two trials, LCIG was found to improve both motor and non-motor symptoms across all subdomains, as measured by the non-motor symptom scale (Antonini et al., 2017; Deuschl et al., 2022). In the GLORIA registry, participants had a mean age of 66 years and a mean PD duration of 13 years. Therefore, results may not apply to patients with more advanced parkinsonism. While studies have demonstrated the beneficial effects of motor and non-motor symptoms on patients with LCIG in advanced stages of Parkinson’s disease, (Kamel and Al-Hashel, 2020) its applicability to most patients nearing the end of life is unlikely. Firstly, obtaining insurance authorization for this procedure might prove unfeasible or challenging when patients are in an inpatient setting and/or under hospice care. Secondly, the approval process generally spans several weeks, and given the constraints imposed by the advanced nature of their condition, this option may not be viable. Lastly, surgical interventions may often misalign with the patient’s goals. Although literature supporting these observations is lacking, these are common pitfalls that the authors have encountered in their clinical practice.

With regards to apomorphine, it was initially licensed in the United Kingdom in 1993 (APO-go®, Britannia Pharmaceuticals Ltd., United Kingdom), which was followed by additional licensure in European and non-Euorpean countries. In the United States, apomorphine subcutaneous infusion, as opposed to apomorphine subcutaneous pen, is not yet FDA-approved (Pietz et al., 1998; Odin et al., 2015). There is insufficient evidence on the safety of apomorphine infusion in PwP at EOL. In the broader context of managing advanced PD, the EAN-ES MDS guidelines suggests offering apomorphine infusion to eligible patients. The recommendation is based on a single randomized clinical trial and a few open label studies (Deuschl et al., 2022). Patients were excluded from the TOLEDO trial if they had atypical parkinsonism, a significant postural instability or orthostatic hypotension, cognitive impairment, or moderate psychosis. Side effects were frequent in the treatment group, including 22% experiencing nausea and somnolence (Katzenschlager et al., 2018). Incidence of neuropsychiatric side effects range was 36–44% and orthostatic hypotension was seen in up to 16% of patients.

##### 5.2.1.2. Group B

LCAS can be used for those who can swallow. Transdermal rotigotine patches can be considered to minimize discomfort and rigidity at EOL when oral intake is not possible, although there might be considerable risks of delirium, agitation and psychosis as previously mentioned (Hindmarsh et al., 2021; Ibrahim et al., 2021).

#### 5.2.2. Non motor symptoms

##### 5.2.2.1. Pain

###### 5.2.2.1.1. Group A

The PD-Pain Classification System (PD-PCS) helps identify PD-related pain and non-PD related pain. Pain can be neuropathic, nociceptive (musculoskeletal, dyskinesia, or dystonic), or nociplastic (neuropsychiatric, central; Mylius et al., 2021). The success in treating pain is determined by identifying the cause. Unfortunately, many medications used in pain management at EOL (e.g., opioids) can worsen many non-motor symptoms of parkinsonism like hypotension, delirium, and constipation, to name a few. Agents like Gabapentin and duloxetine are reasonable pharmacological options for treating neuropathic pain (Djaldetti et al., 2007). For nociceptive pain, first line agents include acetaminophen and non-steroidal anti-inflammatory drugs (Buhmann et al., 2020) though insufficient evidence was found to support the use of oxycodone-naloxone prolonged-release capsules, it was considered potentially useful in a systematic review (Seppi et al., 2019).

If pain is felt to be related to the OFF state or represents a dyskinetic or dystonic wearing off pain, then increasing the levodopa dose may alleviate the pain. The use of adjunctive medications to prolong the ON period may be helpful (Buhmann et al., 2020). Focal, cramping or dystonic pain can respond well to botulinum toxin A injections. Consulting a movement disorder specialist with expertise in injections is recommended. A trial investigated the utility botulinum toxin A in reducing dystonic limb pain in advanced parkinsonism. Injections were found to be safe although pain reduction was not significantly different from placebo (Bruno et al., 2018). In many practices, there are logistic barriers to implementing inpatient neurotoxin injections.

One meta-analysis investigating the overall effectiveness of different therapies in PD pain found a greater pain reduction with safinamide, followed by cannabinoids, opioids, multidisciplinary pain management, catechol-O-methyltransferase inhibitors, electrical and Chinese therapies (Qureshi et al., 2018). Supplementing vitamins B6, B12, and folate helps prevent homocysteine-induced length-dependent peripheral neuropathy that can occur due to peripheral metabolism of levodopa. Some movement disorders experts suggest a single multivitamin that includes these vitamins should be a part of the daily medications for every PwP who is getting levodopa therapy (Ahlskog, 2023).

###### 5.2.2.1.2. Group B

Although opioids have bothersome side effects, such as constipation and somnolence, they are still acceptable at EOL for PwP in this group. Opioids can be particularly useful in managing not only pain in the last days of life, but also shortness of breath. When unable to swallow tablets, alternative methods of administering opioids like syringe drivers have been used with success (Campbell et al., 2010; Butt et al., 2019; Żylicz, 2022).

Concentrated forms of liquid opioids, such as morphine, oxycodone and hydromorphone, are available and can be administered sublingually. Immediate-release opioid tablets can also be administered intrarectally.

##### 5.2.2.2. Dementia and psychosis

###### 5.2.2.2.1. Group A

Psychosis in PwP could reflect disease progression or be a complication of dopaminergic therapies. Optimal management of motor symptoms may be at odds with managing neuropsychiatric symptoms in advanced parkinsonism. There is paucity of controlled studies examining the comparative efficacy and safety of different algorithms for managing acute agitation and psychosis among PwP in-hospital. This section provides general guidance to manage psychosis which may be applicable to inpatient management. Intercurrent illnesses like respiratory and urinary tract infections, constipation, dehydration, electrolyte derangements must be treated promptly. As a next step in managing hallucinations or other psychotic symptoms in PwP who are treated with complex regimens, medications should be reviewed with the aim of reducing or discontinuing potentially offending medications. Anticholinergics should be stopped first, followed by selegiline, dopamine agonists, amantadine, and COMT inhibitors (Friedman and Factor, 2000). Levodopa dose then should be re-assessed. If psychosis persists and further dose reduction will cause bothersome motor impairment, antipsychotics can be added. First generation antipsychotics should be avoided (Żylicz, 2022). Among atypical antipsychotics, quetiapine and clozapine are the preferred agents. Antipsychotics improve the symptoms of psychosis-hallucinations, agitation, and the confusion that may accompany delirium events. They do not improve the confusional state in PwP which is a result of cognitive impairment. Hence, treating these symptoms should be based on the degree of discomfort the patient and the family experience (Friedman and Factor, 2000). Pimavanserin is a novel antipsychotic that received FDA approval in 2016 to manage psychosis and visual hallucinations in PD Psychosis (Cusick and Gupta, 2023). It has shown to reduce hospitalization and mortality rates compared to quetiapine (Sankary et al., 2020; Layton et al., 2023). Although the average age of the patients in two groups were 80, patients that had claims for hospice or palliative care were excluded from both these studies. Currently, it is only available as an oral formulation. Importantly, it is the first antipsychotic without any dopaminergic antagonism and has no significant drug interactions with carbidopa/levodopa (Meltzer and Roth, 2013). Notable contraindications include QT prolongation. While the manufacturer does not does not suggest dose adjustment in renal disease, caution is recommended when treating those with severe renal impairment.

At EOL, many PwP are on medications to manage cognitive impairment. Rivastigmine has been shown to reduce the decline of PD dementia compared to placebo in a 6 month follow up, with maximum efficacy within 3 months. Rivastigmine may also be beneficial for PwP with hallucinations (Zahodne and Fernandez, 2008; Lokk and Delbari, 2012). Common side effects include gastrointestinal symptoms. Donepezil is sometimes used to manage PD dementia. The Movement disorders Society evidence-based review of non-motor symptoms therapeutics rated donepezil as insufficient evidence but potentially useful (Seppi et al., 2019). The most common reported side effect in the PD population with donepezil is psychosis and dizziness along with GI side effects (Ravina et al., 2005). Careful consideration should be made when continuing these medications, especially when concomitant side effects can hamper QOL.

###### 5.2.2.2.2. Group B

Terminal agitation experienced in PwP can be managed with midazolam. Pimavanserin and preferred atypical antipsychotics (clozapine and quitepine) can be safely used in this population. Cholinesterase inhibitors may be discontinued to reduce pill burden (Żylicz, 2022).

##### 5.2.2.3. Gastrointestinal symptoms

###### 5.2.2.3.1. Group A

Nausea, dysphagia, and malnutrition are quite common in advanced PD. When concerns about weight loss and/or swallowing dysfunction emerge, an evaluation by a dietician and/or a speech language pathologist is indicated. In some cases, multi-disciplinary management by gastroenterologists and otolaryngologists is necessary to guide individualized management. A consensus statement on the management of gastrointestinal manifestations of PD was published in 2021 (Schindler et al., 2021). Most patients will benefit from dietary modifications and swallowing maneuvers and exercises. Domperidone is a peripheral D2 dopamine blocker which only crosses the blood brain barrier in minute amounts minimizing risk of aggravating parkinsonism. It can be considered for the management of nausea or dyspepsia in PwP. Available formulations include an oral tablet and a suppository and it can be administered 30 min prior to a meal. Domperidone is not available in the US. Its use is deemed possibly efficacious and supported by Class II-IV evidence per a review for the Movement Disorder society Task Force (Ferreira et al., 2013; Seppi et al., 2019). On the other hand, metoclopramide, cinnarizine and prochlorperazine, some of which are commonly antiemetics in hospice, carry a significant risk of worsening parkinsonism and should be avoided. Ondansetron, a 5HT3 receptor antagonist, is likely beneficial and well tolerated. Interestingly, several studies have also shown benefit of this agent in PD psychosis (Kwan and Huot, 2019). For persistent nausea, dyspepsia or gastric dysmotility despite pharmacological management, referral to gastroenterology should be considered.

Limited data is available on non-invasive brain stimulation for dysphagia. Published studies on transcutaneous electrical stimulation do not suggest swallowing benefit (Schindler et al., 2021). Procedural interventions might be considered for a subset of patients under the supervision of the multidisciplinary team. With data from Class IV evidence, neurotoxin injection to the cricothyroid muscle, by an experienced clinician, may be an option to manage those whose swallowing dysfunction is primarily related to upper esophageal spasm (Schindler et al., 2021). Nutritional modifications should take into account both the safety aspects as well as nutritional status. Patients with advanced PD receiving LCIG were reported to experience improvements across many non-motor symptoms including gastrointestinal symptoms although weight loss was reported in 6.7% of patients in one large registry (Abbruzzese et al., 2012; Antonini et al., 2017; Kamel and Al-Hashel, 2020). There are no specific recommendations for percutaneous endoscopic gastrostomy (PEG) placement in PD. Similar to other conditions, experts suggest PEG can be considered when severe swallowing dysfunction lasts more than 4 weeks resulting in weight loss (Schindler et al., 2021). Decisions should be individualized, taking into account the patient’s perspective, prognosis and overall medical status. For PwP who receive LCIG, enteral nutrition via the gastric port can be provided when needed while monitoring for possible changes in medication absorption.

###### 5.2.2.3.2. Group B

Procedural interventions for dysphagia and PEG are unlikely to help in this population.

## 6. Conclusion

In conclusion, this study sheds light on the complex and often challenging landscape of providing care to patients with Parkinson’s disease (PD) near the end of life (EOL). The study touched on underutilization of PC resources for this population despite the high burden of comorbidities and frequent hospital admissions in the last year of life. Majority of the patients were full code, and less than majority of the patients were seen by PC. Patients who passed in the hospital had a higher health care resource utilization (invasive procedures, ICU transfers) and lower PC involvement. The findings highlight the need for improved integration of palliative care (PC), particularly in the inpatient setting. The delayed involvement of PC services and the mismatch between patient/family expectations and providers’ goals of care highlights the importance of early recognition of declining health trajectories and initiating conversations about advance care planning. The study also highlights the complexities of medication management for inpatient PD patients. Frequent deviations from home medication regimens in both EOL and non-EOL group, included underdosing. Fortunately, giving contraindicated medications and missing medications for PD were uncommon errors.

Additionally, efforts to develop standardized guidelines for PC integration, advance care planning, and medication management for patients with advanced PD at EOL are essential to improve patient outcomes and quality of care.

## Data availability statement

The raw data supporting the conclusions of this article will be made available by the authors, without undue reservation.

## Ethics statement

The studies involving humans were approved by Cleveland Clinic Institutional Review Board, IRB # 21-1128. The studies were conducted in accordance with the local legislation and institutional requirements. Written informed consent for participation was not required from the participants or the participants’ legal guardians/next of kin in accordance with the national legislation and institutional requirements.

## Author contributions

SB: Data curation, Methodology, Conceptualization, Formal analysis, Visualization, Writing – Original draft, Writing – Review & editing. EA: Data curation, Methodology, Writing – Original draft, Writing – Review & editing, Formal analysis, Visualization. JY: Data curation, Writing – Review & editing. NM, CS, OH: Formal analysis, Writing – Review & editing. BW: Writing – Review & editing. RS, AM: Conceptualization, Supervision, Visualization, Writing – Review & editing.

## Funding

The author(s) declare that no financial support was received for the research, authorship, and/or publication of this article.

## Conflict of interest

BW serves as Section Head of Movement Disorders at the Cleveland Clinic. Over the years, he has received research grants from the NIH and Parkinson’s Foundation. He has served as site investigator and /or co-investigator for clinical research studies sponsored by grants from Industry to Cleveland Clinic. Currently, he site PI for Neuroderm (Mitsubishi Tanabe Pharma), Discern (Great Lakes Neurotech/NIH R44), Tempo2, and Tempo3 studies (Cerevel Therapeutics) and a grant from the Parkinson’s Foundation to improve the inpatient care of People with PD. He has served as a consultant or speaker for Medtronic, Boston Scientific, and Abbott for less than $5,000 in the last year.

The remaining authors declare that the research was conducted in the absence of any commercial or financial relationships that could be construed as a potential conflict of interest.

The author(s) declared that they were an editorial board member of Frontiers, at the time of submission. This had no impact on the peer review process and the final decision.

## Publisher’s note

All claims expressed in this article are solely those of the authors and do not necessarily represent those of their affiliated organizations, or those of the publisher, the editors and the reviewers. Any product that may be evaluated in this article, or claim that may be made by its manufacturer, is not guaranteed or endorsed by the publisher.
